# Sexually transmitted infections—Research priorities for new challenges

**DOI:** 10.1371/journal.pmed.1002481

**Published:** 2017-12-27

**Authors:** Nicola Low, Nathalie J. Broutet

**Affiliations:** 1 Institute of Social and Preventive Medicine, University of Bern, Bern, Switzerland; 2 Department of Reproductive Health Research, World Health Organization, Geneva, Switzerland

## Abstract

In an Editorial, Guest Editors Nicola Low and Nathalie Broutet discuss the Collection on sexually transmitted infections in the context of research priorities in the field.

The World Health Organization (WHO) estimates that more than 1 million new sexually transmitted infections (STIs) are acquired each day [1]. STIs are pernicious players in the global burden of disease, their management stymied by the diversity of pathogens, social stigma, and commonly mild or nonexistent symptoms. As quietly as they persist, STIs have prominent sequelae—for example, roughly one-third of pregnant women infected with syphilis experience adverse birth outcomes including stillbirth, human papillomavirus (HPV) infection leads to an estimated 266,000 cervical cancer deaths annually, and some bacterial STIs cause pelvic inflammatory disease, female infertility, preterm delivery, and low birthweight. The imperative for innovation at this time is one of the strategic directions of the WHO global health sector strategy to address the burden of STIs [2].

This month, *PLOS Medicine* launches the research content from our Collection on Prevention, Diagnosis, and Treatment of STIs. The Collection will feature Research Articles submitted in response to our call for papers this past summer, with related Perspectives from international STI experts. Two pressing themes frame current research in this area. First, the means of prevention for HIV and for other STIs are now decoupled, with gonorrhoea and syphilis amongst men who have sex with men on the rise as an unintended consequence of antiretroviral therapy that renders HIV undetectable in blood, and of the availability of pre-exposure prophylaxis (PrEP) [3]. When condoms were the main prevention technology for all sexually transmitted pathogens, prevention messages were unified but uptake was patchy. PrEP, on the other hand, is now being adopted and adhered to more readily by the people at highest risk of acquiring HIV infection, but does not prevent any other STI. In anticipation of increases in risky sexual behaviours in the context of PrEP, researchers and practitioners should actively promote primary STI prevention, including promotion of barrier methods. Indeed, secondary prevention strategies, including more frequent screening for STIs, are not a panacea because an increased rate of untargeted treatment can drive antimicrobial resistance (AMR) [4].

Second, modern STI management is being increasingly challenged by AMR, which has already compromised the treatment of gonorrhoea [5] and is expanding geographically. Some possible solutions to the threat of AMR are explored in the Collection. In their mathematical modelling study, Xavier Didelot and colleagues project how cautious use of previously abandoned antimicrobials could mitigate the spread of resistance [6]. A linked Perspective by Magnus Unemo and Christian Althaus discusses the study in the context of current knowledge about gonococcal resistance to cephalosporins [7]. Additionally, risk assessment of the impact on AMR should likely be required before the introduction of new preventive strategies or guidelines. For example, new molecular diagnostic tests for *Mycoplasma genitalium*, which is under-recognised as a cause of urethritis and cervicitis, are considered likely to worsen already alarming levels of resistance to macrolide and fluoroquinolone antimicrobials [8]. Syndromic treatment of symptomatic urethritis, the norm in both high- and low-income settings, has actually limited the use of antimicrobials. Paradoxically, improved aetiological diagnosis will result in increased treatment, and multidrug resistance, because asymptomatic infections will also be detected and treated. New guidelines should recommend diagnostic test and treatment strategies for urethritis and *M*. *genitalium* that minimise the risk of AMR.

Challenges like these require real-world knowledge, and we believe that insights from social science methodology are critical for illuminating possible solutions. We are delighted that this Collection includes a qualitative study from Kipruto Chesang and colleagues, in which the authors describe many of the challenges that healthcare providers (HCPs) worldwide face in delivering STI care [9]. The authors interviewed 87 HCPs working in HIV care centres across Kenya. Their analysis shows strong HCP commitment to the provision of high-quality STI care but underscores the impact of stigma and culturally embedded gender roles. This study suggests that clinics often do not provide for the sexual and reproductive health needs of men and boys, even though their active engagement is essential for the sexual health of both women and men [10]. Chesang and colleagues also describe the day-to-day health service barriers of antimicrobial treatment failure, ascribed to resistance, insufficient training, and drug stock-outs. In relation to the last point, Collection authors Stephen Nurse-Findlay and colleagues explore the origins of a vexing worldwide shortage of benzathine penicillin for the treatment of maternal syphilis, using country-level surveys and stakeholder interviews [11]. They find that local stock-outs are not just the result of demand-side under-procurement, but of supply-side inflexibility and market exits for this cheap, off-patent drug.

In more auspicious developments, digital technologies and newer diagnostics with simple requirements for specimen collection and transport are driving innovations in access to STI care. In this area, Collection authors Emma Wilson and colleagues evaluated the benefits of providing “e-STI testing and results” in a randomised controlled trial done in London, UK [12]. They used text messages to invite people to place an online order for self-sampling kits for chlamydia, gonorrhoea, syphilis and HIV. The e-STI testing and results intervention increased the proportion of people tested for STIs, and slightly increased the proportion diagnosed with any STI, compared with people sent a simple text message with information about the location of STI clinics. The researchers used multiple active methods to reach and engage their target group; therefore, to sustain the benefits of the e-STI testing intervention, these health promotion activities would need to continue. Even in times of economic austerity, e-STI testing should not be seen as a substitute for fully funded clinic-based services [13].

Meanwhile, highly efficacious vaccines against human papillomaviruses and hepatitis B virus have demonstrated the benefits of innovation in vaccine development, and results in this Collection suggest that further innovation will not be wasted. Christine Johnston and colleagues’ findings support the development of a vaccine against herpes simplex type 2 (HSV-2) as the next most promising vaccine priority [14]. In people with HSV-2 antibodies enrolled in epidemiological studies in the Americas and sub-Saharan Africa, just 3.7% had prevalent infection with more than one HSV-2 strain, indicating the effectiveness of naturally occurring protection.

Future progress in understanding the pathogenesis of STIs in women, who bear a large proportion of the world population’s burden of STIs, will rely on the innovations of high-throughput molecular sequencing methods that have revealed the complexity of the vaginal microbiome. In a Perspective, Janneke van de Wijgert discusses what we now know about interrelationships between exogenous sexually transmitted bacterial pathogens, dysbiosis affecting the lactobacillus-dominated microbiome, and pathobionts, commensal bacteria with pathogenic potential [15]. However, improved understanding of the nature and properties of vaginal microbiomes will be required for the development of approaches for optimising vaginal health.

Successes in STI control require commitments to addressing the economic, social, cultural, and behavioural determinants of STIs. In the face of a widening spectrum of infectious agents that can be transmitted through sexual contact, as described in an Essay by Kyle Bernstein and colleagues, interdisciplinary action will be important to the development of effective interventions [16]. High-quality research is one of the solutions that, together with strengthened capacity, promotion of sexual rights and political commitment, can secure a future of effective STI prevention, diagnosis, and treatment.

## Abbreviations

AMR: antimicrobial resistance
HCP: healthcare provider
HSP-2: herpes simplex type 2
PrEP: pre-exposure prophylaxis
STI: sexually transmitted infection
WHO: World Health Organization
